# Food Emotional Perception and Eating Willingness Under Different Lighting Colors: A Preliminary Study Based on Consumer Facial Expression Analysis

**DOI:** 10.3390/foods14193440

**Published:** 2025-10-08

**Authors:** Yuan Shu, Huixian Gao, Yihan Wang, Yangyang Wei

**Affiliations:** 1 Architecture and Design College, Nanchang University, Nanchang 330031, China; 2 School of Art, Wuhan Business University, Wuhan 430056, China

**Keywords:** food, lighting, color, appetite, emotional perception, generalized facial expression recognition

## Abstract

The influence of lighting color on food is a multidimensional process, linking visual interventions with people’s perception of food appearance, physiological responses, and psychological associations. This study, as a preliminary exploratory research, aims to initially investigate the effects of different lighting colors on food-induced consumer appetite and emotional perception. By measuring consumers’ physiological facial expression data, we verify whether the results are consistent with self-reported subjective evaluations. Questionnaires, Shapiro–Wilk tests, and one-sample *t*-tests were employed for data mining and cross-validation and combined with generalized facial expression recognition (GFER) technology to analyze participants’ emotional perceptions under various lighting colors. The results show that consumers displayed the most positive emotions and the highest appetite under 2700 K warm white light. Under this condition, the average intensity of participants’ “happy” emotion was 0.25 (SD = 0.12), indicating a clear positive emotional state. Eating willingness also reached its peak at 2700 K. In contrast, blue light-induced negative emotions and lower appetite. Among all lighting types, blue light evoked the strongest “sad” emotion (M = 0.39). This study provides a preliminary exploration of the theoretical framework regarding the relationship between food and consumer behavior, offering new perspectives for product marketing in the food industry and consumer food preference cognition. However, the generalizability of its conclusions still requires further verification in subsequent studies.

## 1. Introduction

The color of food plays a crucial role in consumers’ food selection, serving as one of the most important cues perceived before tasting. The association between color and taste has been studied throughout human evolution [1]. Variations in color directly affect both physiological and psychological perception. The influence of color on taste perception and consumption behavior is regulated by emotions; food color can alter consumers’ brain perception and may either enhance or suppress appetite. For example, most people associate the taste of salt with white and blue, sweetness with red and pink, sourness with green and yellow, and bitterness with brownish-black and violet [2]. Spence further proposed that the influence of color on taste is not a singular pathway but is achieved through ‘cross-modal correspondence’—red not only makes food appear sweeter but also enhances the nasal sensitivity to sweet-related smells. This multi-channel synergistic effect amplifies the impact of color on appetite [3]. Additionally, Spence suggested that cultural background significantly reshapes color–taste associations. In a test involving a transparent blue beverage, Taiwanese participants perceived it to have a mint flavor, while British participants thought it would taste like raspberry. This indicates that due to differences in food markets across regions, people develop distinct color–taste associations in their daily lives. For instance, in Taiwanese food culture, blue foods or drinks with mint flavor may have left a strong impression, while in the UK, blue foods or related advertisements associated with raspberries may have led the public to associate the color blue with raspberry flavor. Different lighting color temperatures can change the surface appearance of food, thereby influencing participants’ perception and acceptance [4]. For example, when tasting soda and mineral water, consumers tend to actively choose blue among red, green, and blue options [5]. Before tasting, people perceive objects as food through the visual system and predict their taste before deciding whether to purchase or consume. Thus, color is often the most decisive factor in food choice and evaluation.

Consumers tend to prefer brightly colored foods, though the degree of preference depends on the inherent color of the food itself. They typically show stronger preferences for red, yellow, and green foods, and weaker preferences for colorless or magenta foods. The remembered or expected color of food can significantly influence consumers’ preference for its vividness [6]. Under different colored lighting, the eyes transmit visual images to the brain, where different light frequencies are interpreted as different colors, thereby affecting appetite and dietary decisions [7]. Food color cues also alter consumers’ chemosensory perception [8,9]. There seems to be a variety of possible relationships between color and taste, with emotions also playing a role as intermediators. The hue and saturation of food or beverage colors influence the expectations formed in the taster’s mind, and different individual or group consumers may associate specific colors with different tastes [10]. Moreover, lighting color and temperature influence shoppers’ quality perception of products [11]. When ambient lighting is red or blue, consumers display stronger purchasing tendencies than under green or white lighting, and they are willing to pay higher prices.

The visual characteristics of food are difficult to evaluate, as they can change over time due to the natural variations in the product and the differing consumer perceptions caused by factors such as lighting [12]. The lighting environment surrounding food can significantly alter consumers’ appetite and emotional perception. As a subtle environmental factor, ambient lighting is essentially nonrestrictive and is therefore unlikely to encounter consumer resistance [13]. Food under different colored lighting can elicit distinct emotional responses and shape consumers’ emotional perception of the food. The original surface color of food may change under colored lighting, which may in turn regulate consumers’ motivation to consume [14]. Increasing the diversity of food colors can enhance consumption, but when we observe abnormal colors (or those perceived by consumers as abnormal), it can lead to the inhibition of appetite behavior [15]. For instance, acceptance of meat color differs depending on illumination source—incandescent, fluorescent, or metal halide lighting. Compared with fluorescent and metal halide lamps, consumers perceive beef color more favorably under incandescent lighting [16]. However, for poultry meat products, from a food safety perspective, lighting alters people’s perception of doneness and their willingness to consume meat patties. Certain energy-saving lights, such as LED and halogen lights, can make meat patties appear more cooked than they actually are, thus increasing the willingness to eat undercooked meat patties. This finding reveals the ‘dual risk’ of lighting color: it can not only mislead consumers in their judgment of freshness but also obscure potential food safety risks (such as undercooked meat), a risk that is particularly prominent in contexts like fast food, where visual cues are relied upon to judge doneness [17]. Warm lighting may enhance the appeal of certain dishes, whereas cooler lighting may make others appear fresher. The interaction between food and lighting is essential for creating dining atmospheres that enhance sensory experiences.

Under fluorescent lighting, consumers exhibit the largest number of subconscious responses, while metal halide lighting triggers the strongest emotional reactions. In contrast, halogen lighting produces the most attractive illumination effects in conscious perception, while metal halide and LED lights are perceived as least attractive. Furthermore, when lighting conditions change, the right hemisphere of the brain—associated with emotional responses—shows greater engagement, indirectly reflecting the impact of lighting environments on emotional perception [18]. One study found that participants’ willingness to consume bell peppers was highest under yellow lighting and lowest under blue lighting. Compared with bright environments, darker settings reduced both liking for bell pepper appearance and willingness to consume [19]. Similarly, apples under white or yellow light were rated as more desirable and elicited stronger consumption willingness, with a particular preference for apples under yellow light. Conversely, apple flavor intensity was weaker under blue light than under yellow, white, or red light [14]. For cakes, aligning icing and body color with red illumination enhanced visual appeal and appetite [7]. This may be because red light reduces feelings of calmness, relaxation, stability, and pleasure while intensifying irritability and tension. Blue light reduces relaxation and stability while increasing irritability; green light decreases pleasantness, whereas yellow light can reduce irritability [20].

To examine consumer food preferences and emotional perception under different lighting, common research methods include facial expression analysis, electroencephalography (EEG), and eye-tracking. Combining Spence’s cross-modal correspondence theory and Maughan’s food safety perspective, these approaches can not only capture the impact of lighting color on the ‘subjective perception’ of participants but also offer potential avenues for further research into the mechanisms linking light color to ‘objective food attributes’ (such as freshness and doneness). For example, one study used facial expression analysis to assess consumer acceptance of liqueur [21]. Eye-tracking technology objectively records eye movements and changes in visual attention during decision-making, thereby enriching datasets of consumer food behavior [22]. Research has shown that food color affects visual fixation, with bright colors attracting more attention [23]. Heat maps and gaze trajectories derived from eye-tracking experiments confirm that food color influences the focus and attention of visual perception, and people rely on first impressions to infer taste, thereby shaping psychological needs [24]. EEG experiments also revealed that red light (5200 K) increased participants’ late positive component (LPC) amplitudes, thus enhancing appetite, whereas green light reduced appetite, particularly under food-related visual stimuli [7]. Consequently, implicit measurements may be more suitable than explicit ratings for capturing the complexity of food experiences in real-life contexts [25].

Implicit measures indirectly monitor consumers’ food experiences through central nervous system activity (e.g., EEG, MRI), autonomic nervous system responses (e.g., skin conductance, heart rate), and expressive behaviors such as facial expressions. In one study of eating speed and food preference, implicit measures (facial expressions and heart rate) indicated faster eating and stronger emotional reactions at home compared to in a laboratory setting, including expressions such as “happy,” “angry,” and “disgust.” Explicit measures included liking scores and ratings of sensory attributes, whereas implicit measures included facial expressions, heart rate, and consumption duration measured by facial recognition software. The results concluded that implicit measures are more sensitive to consumer responses than explicit measures [26].

Facial expressions, as one form of implicit measurement, are not only a universal means of conveying emotion but also provide feedback on individual emotional states. Among implicit measures, facial expressions are among the most specific indicators of emotion and can be easily obtained using video recording [27]. For instance, Face Reader (FR 330) software has been applied to evaluate consumer acceptance of meat products from both sensory and no-sensory perspectives [28]. In the context of food consumption, facial expressions reveal immediate emotional reactions to sensory properties such as taste, texture, and appearance. By directly capturing participants’ facial expressions while consuming food and quantifying their basic emotions within a defined timeframe, researchers can evaluate consumer emotional states [29].

The Facial Action Coding System (FACS) enables objective measurement of consumer facial expressions, identifying specific emotions associated with food experiences. By recording unconscious facial expressions during tasting, researchers capture emotional details without requiring participants to self-report emotion terms. Facial expression recognition technology classifies facial expressions during tasting to measure unconscious consumer emotions [30]. Thus, programs like Face Reader can analyze emotions expressed during food consumption [31]. Studies show that facial recognition technology provides greater sensitivity than hedonic scales, as self-reported measures may be influenced by memory or prior associations [32]. This highlights the close relationship between food preferences and emotional responses. The diversity and continuity of implicit measures offer deeper perspectives on consumer responses to food [23]. Consumer emotions not only represent positive or negative evaluations of stimuli but also reflect contextual and social evaluations of emotional experiences [33].

Facial expression recognition, based on computer vision and machine learning, can accurately identify and analyze emotional information conveyed by facial expressions. Once a face is detected, emotion features are extracted [34]. The primary task is to classify facial images into correct expression categories [35], largely by recognizing muscle movement patterns and separating expression features from facial identity features [36]. Using high-resolution cameras, facial recognition systems capture facial images, extract feature points via image processing algorithms, and analyze facial expression patterns in video data [37]. For example, a raised mouth corner and crow’s feet at the eye corners are typically associated with “happy,” while frowning eyebrows and closed lips may indicate “angry” or “disgust”.

Compared with traditional methods such as self-report surveys, facial expression recognition offers significant advantages. Self-reports are inevitably influenced by psychological and social factors, reducing data accuracy and reliability [38]. In contrast, facial recognition objectively captures expressions in real time, avoiding participant bias and providing more authentic data. Expression parameters may vary with video duration [39], and different emotional peaks can appear at different moments, underscoring the importance of temporal dynamics in food-related emotions [40]. To overcome biases inherent in self-reported sensory terms, facial expression recognition adopts an unconscious approach, recording expressions automatically [30].

For instance, a study comparing self-reports and implicit measures of beer tasting found that self-reports displayed the highest discriminative ability [41]. Explicit measures reflect overall preferences, which may depend on expectation, familiarity, and sensory characteristics, but lack detailed temporal tracking. Face Reader software can supplement this by analyzing emotion responses and rationalizing observed emotions [42]. Thus, combining implicit (facial expression) and explicit (scoring) measures provides complementary insights [43]. In explicit measurement, the Likert scale is often used to gather subjective ratings of food preference and eating willingness under different lighting conditions. This quantitative approach complements the qualitative strengths of facial expression analysis and offers a more comprehensive understanding of consumer responses.

In this study, generalized facial expression recognition (GFER) technology was used to evaluate consumers’ emotional perception of food. At the same time, subjective preference ratings were collected via a Likert scale under different lighting conditions. This approach examined whether subjective preference scores correlated positively with facial expression recognition results. By combining subjective self-reports with objective GFER analysis, the study sought to cross-validate results. Using fried chicken and French fries as sample foods, the study investigated the influence of colored lighting on consumer preference and analyzed emotional state changes during observation. Facial recognition was conducted through multi-view face detection based on deep convolutional neural networks, enabling quantification and encoding of facial features [34]. The aim is to preliminarily explore consumer preferences and emotional perceptions of food under different lighting colors and further investigate the application value of facial expression recognition technology in assessing consumer food preferences. The study aims to reveal consumers’ food preferences and emotional perceptions under different lighting colors and further highlight the application value of facial expression recognition and analysis technology in assessing consumers’ food preferences.

Accordingly, the following hypotheses were formulated:

1. Adjusting consumer preferences through lighting color can potentially influence consumers’ eating behavior and food choices.
2. By controlling the changes in light color, participants experienced significant emotional fluctuations towards food under different lighting environments.
3. In the context of food consumption, consumers’ facial expressions can reflect their emotional state type and intensity of emotional fluctuations towards food, and are positively correlated with the results of subjective preference surveys.

## 2. Materials and Methods

### 2.1. Experimental Setup

This study, as a preliminary exploratory research, recruited 18 voluntary participants (aged between 18 and 65 years) to assess appetite ratings and conduct facial expression analysis under 6 different lighting conditions, ultimately obtaining 108 sets of valid data. The experiment recruited 18 volunteer participants aged between 18 and 65 years. Participants were required to have no medical history that could affect food perception, such as diabetes or cardiovascular disease. All participants completed an olfactory identification test and a taste identification test to evaluate their sensitivity to basic tastes and ensure normal sensory function. In addition, color vision was screened using the standard Ishihara color blindness test to exclude individuals with color vision deficiencies, ensuring accurate discrimination of lighting colors.

Before the experiment, participants completed a hunger scale to control for the influence of hunger levels on food perception. The experiment was conducted under similar hunger conditions to minimize interference. Participants also reported their general liking of fried chicken and French fries to assess baseline preferences. Participants were required to taste food samples under different color temperature lighting conditions, and their facial expressions, personal likes, and willingness to eat scores were recorded.

The food samples used in the experiment were high-calorie foods, specifically fried chicken and French fries. All samples were purchased from the same restaurant and prepared within 15 min prior to the start of each session to ensure freshness and consistent taste. Food portions were standardized to maintain experimental consistency and reproducibility.

Six different color temperature lighting conditions were used in the experiment to simulate various lighting environments. Specifically, these included 2700 K (warm white light), 4000 K (neutral white light), 6500 K (cool white light), red light, green light, and blue light. LED lighting ensured stability and uniformity. The selection of color temperatures was based on their common application in commercial and domestic settings and their potential influence on food visual perception.

Participants’ facial expressions under different lighting conditions were recorded using a high-definition camera, positioned 1.5 m from the participants to capture their full facial expressions clearly. The camera resolution and frame rate were set to maximum for optimal image quality. Facial expression data were analyzed using Generalizable Facial Expression Recognition (GFER) technology, which integrates image synthesis and deep metric learning by generating expression sets of identical identities for comparison and classification [44].

All experimental equipment was calibrated prior to data collection. Camera and lighting positions were carefully adjusted according to the experimental design. Environmental temperature and humidity were also controlled within appropriate ranges to minimize external interference.

### 2.2. Experimental Procedure

#### 2.2.1. Measurement with GFER

**Figure 1:**
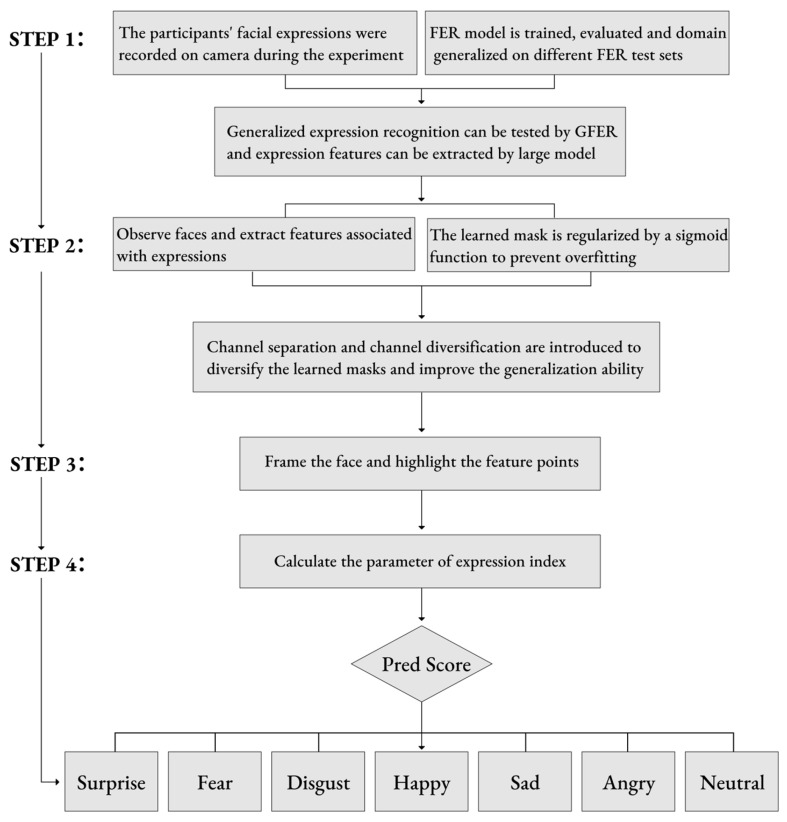
Workflow of facial expression analysis.

The experiment used a high-definition camera to record the facial expressions of participants while tasting food, and used GFER (Generalizable Expression Recognition) to measure feelings in order to capture rapidly changing emotions and target the subconscious part of emotional experience [39]. In the experiment, the camera was set to shoot 1080 p high-definition video to ensure that subtle changes in facial expressions could be accurately captured by the camera. The video data shot in the experiment was used for subsequent facial expression analysis to assess the emotional state of the participants through expression recognition. Generalizable facial expression recognition was implemented in the Python (v3.9.20) programming language, and the framework of the CAFE method was proposed. A fixed pre-trained large model CLIP was used to extract fixed facial features. The trained FER model generated a mask that was used to select features related to facial expressions from the facial features of the subject and make decisions based only on the selected facial features (Figure 1).

After tasting under each lighting condition, participants immediately reported their liking and willingness to eat using a 9-point Likert scale (1 = “dislike extremely”, 9 = “like extremely”). Data collected included both facial expression video and subjective ratings. Expression data were processed using automated software, while rating data were analyzed with statistical software. Descriptive statistics summarized the scores, and Shapiro–Wilk tests were used to test normality before conducting variance analyses and one-sample *t*-tests.

#### 2.2.2. Stimuli and Presentation

The experiment was conducted in a soundproof laboratory with controlled lighting to minimize external disturbance. Participants were informed of the procedure and signed informed consent forms. The study posed no risks to vision or health and was unrelated to commercial interests.

Participants were instructed not to eat within two hours before testing to ensure normal appetite. Participants were subjected to six different lighting test conditions, with each condition corresponding to a lighting environment of a specific color temperature (2700 K warm white light, 4000 K neutral white light, 6500 K cool white light, red light, green light, blue light). These 6 lighting conditions cover representative cool colors (blue, green), warm colors (red, orange, yellow), and neutral colors (white), constructing a complete wavelength gradient from short to long waves while also incorporating typical colors with emotional symbolic significance in visual perception. This systematic variable gradient design allows the study to comprehensively capture the differentiated impact of various color lighting on food perception, thus avoiding misjudgment of trends due to a singular or incomplete variable coverage. Illuminance levels ranged between 9.0 and 13.5 lux. The selection of this brightness range is based on several considerations, the brightness level of 9–13.5 lux is sufficient to simulate common indoor lighting conditions in real life, providing a balance between being neither too dim to make color perception difficult nor too bright to affect the accuracy of color perception. Research has shown that excessively high brightness can lead to participants becoming overly sensitive to colors or feeling discomfort, while excessively low brightness may not effectively present the contrast of different colors. Therefore, this range helps maintain the naturalness and consistency of color perception. Finally, there is an interaction between brightness and color temperature. High color temperatures (e.g., 6500 K cool white light) may require lower brightness levels to avoid glare, whereas low color temperatures (e.g., 2700 K warm white light) can provide a comfortable visual effect at relatively lower brightness levels. By selecting this brightness range, we can more precisely capture the impact of color under different lighting conditions on food perception, while ensuring the applicability and practicality of the experimental conditions. To conceptualize participants’ color perception, chroma (saturation) and hue angle values was calculated as

$$\mathrm{Chroma}=(a^{2}+b^{2})\frac{1}{2}$$

A higher chroma value indicates greater color vividness compared with lower values.

$$\mathrm{Hue}\;\mathrm{angle}\;\mathrm{value}=\tan-1\left(\frac{a}{b}\right)$$

**Figure 2:**
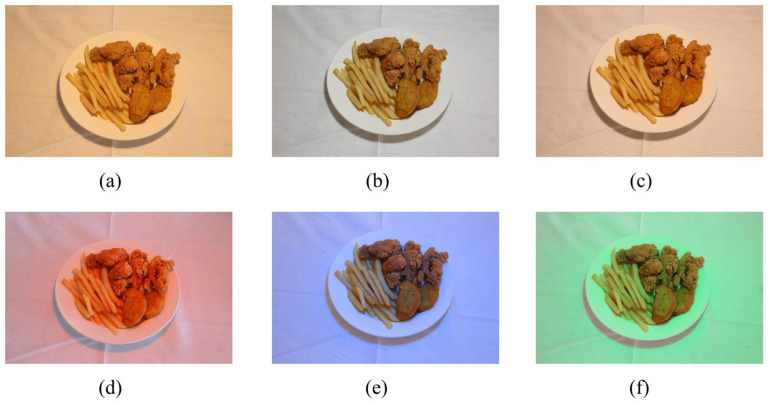
Food samples under different colored LED lighting ((**a**): 2700 K; (**b**): 6500 K; (**c**): 4200 K; (**d**): red; (**e**): blue; (**f**): green).

In this experiment, hue angles of 0°, 90°, 180°, and 270° corresponded to red, yellow, green, and blue, respectively. Under each lighting condition, participants were guided into the experimental area and instructed to remain relaxed and naturally express their feelings toward the food. Fried chicken and French fries were used as food samples, placed on white porcelain plates, and presented under the designated lighting conditions in Figure 2.

After tasting, participants reported their personal liking using a 9-point Likert scale (1 = “dislike extremely”, 9 = “like extremely”). Each lighting condition was presented for 10 s, with approximately 10-s intervals between conditions. Participants were given short breaks between tests to minimize taste fatigue and ensure accurate feedback in subsequent sessions. Facial expression videos and rating data were recorded simultaneously to guarantee consistency and accuracy. In addition, environmental parameters were monitored during the experiment to control potential external influences.

This procedure allowed assessment of participants’ sensory perception of food under different lighting conditions and evaluation of consumer liking and appetite for the tested samples [28].

#### 2.2.3. Experimental Implementation

The HD camera was fixed at eye level, 1.5 m from the participants, to ensure clear capture of facial expressions. All sessions took place in the same room under controlled temperature (20 °C) and baseline fluorescent white lighting (average ± SD = 646 ± 16 lux).

**Figure 3:**
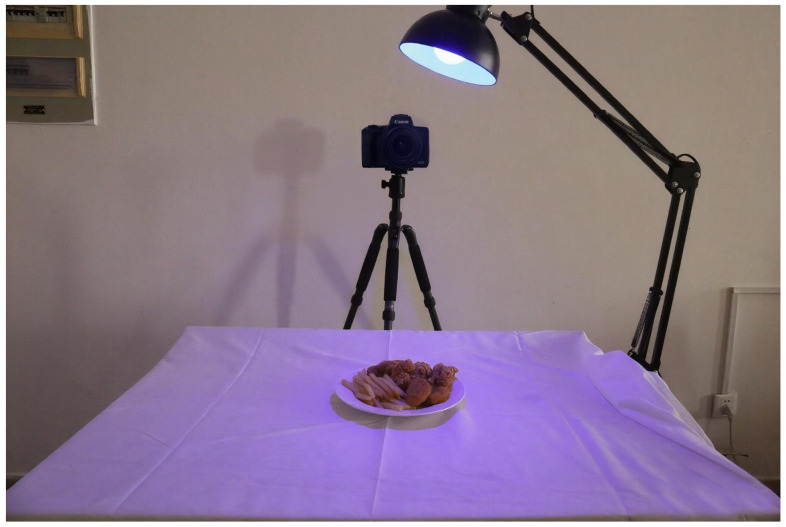
Experimental environment.

Under the six lighting conditions, participants were provided with food samples of the same size and were given one minute to observe the food (Figure 3).

#### 2.2.4. Data Processing and Analysis

Statistical analysis of experimental data was conducted using Generalizable Facial Expression Recognition (GFER) technology to evaluate the influence of different lighting conditions on consumers’ emotional perception.

Step 1. Given an image x from $D_{train}$, the study first used CLIP to extract facial features, denoted as $F\in R^{N\times C}$, where N is the number of images and C is the feature dimension. Furthermore, the learned mask was regularized to ensure generalization to unseen test samples. A sigmoid function was applied to Μ, producing the normalized mask ${Μ}_{s}$.

$${Μ}_{s}=Sigmoid\left(Μ\right)$$

Subsequently, the normalized mask ${Μ}_{s}$ was used to select facial features relevant for expression recognition.

$$\tilde{F}={Μ}_{s}F$$

The classification loss was computed between the selected features $\tilde{F}$ and the corresponding labels y.

$$l_{cls}=-\frac{1}{N}\sum_{i=1}^{N}\left(\log\frac{e^{W_{y_{i}}{\tilde{F}}_{i}}}{\sum_{j}^{L}e^{W_{j}{\tilde{F}}_{i}}}\right)$$

Here, ${\tilde{F}}_{i}$ denotes the selected features of image ${Χ}_{i}$, $W_{y_{i}}$ represents the fully connected (FC) layer weight corresponding to class $y_{i}$, and $y_{i}$ is the label of image ${Χ}_{i}$ [36].

**Figure 4:**
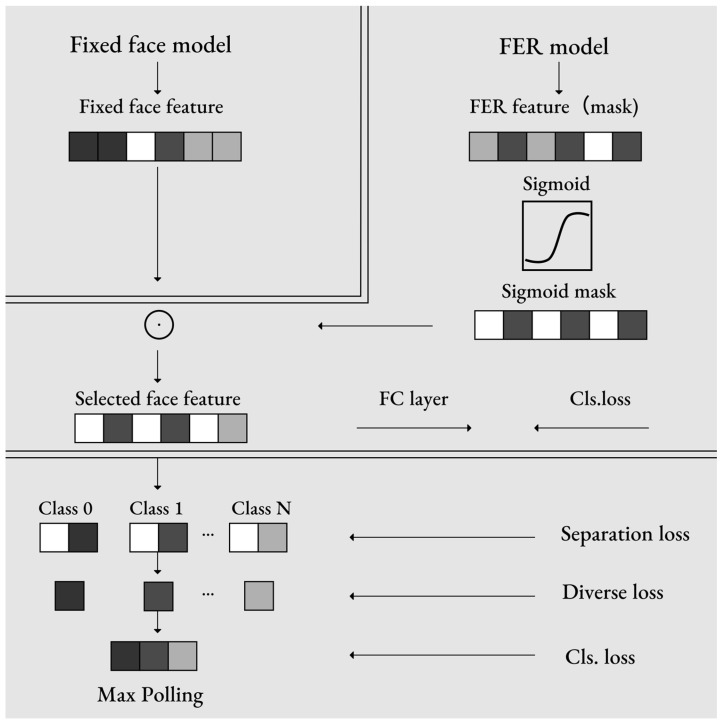
Schematic diagram of Generalizable Facial Expression Recognition.

The experiment first extracted participants’ facial features in order to eliminate domain-related features. Expression-related features were selected from the overall facial features, and decisions were made solely based on these selected features. The variability of emotional states was evaluated by analyzing the probability distribution of these states, as shown in Figure 4.

Step 2. The liking and willingness-to-eat scores under different lighting conditions were subjected to normality testing using the Shapiro–Wilk test (appropriate for small sample sizes ≤ 5000). After confirming normality, a one-sample *t*-test was applied to determine whether *p*-values reached significance (*p* < 0.05). If significant, mean values were compared with test values to analyze the magnitude of differences.

Step 3. An independent-samples *t*-test was conducted to calculate mean emotional scores for participants under different lighting conditions [39]. The data were first tested for normality, followed by a homogeneity of variance test. If *p* ≥ 0.05 (equal variance), a standard independent-samples *t*-test was used; if *p* < 0.05 (unequal variance), Welch’s *t*-test was employed. Based on the *p*-values, if *p* < 0.05, the null hypothesis was rejected, indicating significant differences in liking and willingness-to-eat scores under different lighting colors. If *p* ≥ 0.05, the null hypothesis was not rejected, suggesting insufficient evidence for significant differences.

## 3. Results

### 3.1. Preliminary Response Data from GFER

**Figure A1:**
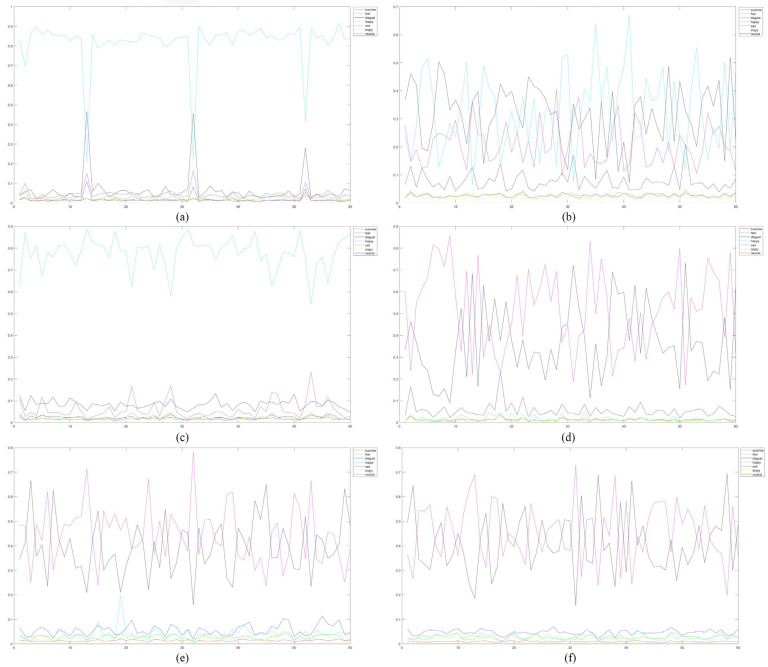
Participants’ emotional intensity under different lighting conditions ((**a**): 2700 K; (**b**): 6500 K; (**c**): 4200 K; (**d**): red; (**e**): blue; (**f**): green) (*x*-axis: duration in seconds).

Analysis of the preliminary response data obtained from the experiment revealed that the fluctuation ranges of consumers’ specific types of emotions varied. In the experiment involving 18 participants and six lighting conditions, the neutral state was the most frequently expressed emotion, as shown in Figure A1. The “neutral” emotion accounted for the highest proportion among all emotion types; however, under stimulation from different colored lighting, the expression of specific emotions increased or decreased accordingly, thereby altering the proportion of neutral states. Time-series data highlighted the average levels of each measured emotion across the entire duration [45]. Both sensory perception and facial responses contributed jointly to these patterns [46]. From the structure of emotional intensity, significant changes in specific emotional states were observed under 4200 K and 2700 K lighting. In particular, the “happy” emotion fluctuated around a level of 0.8 ± 0.25, standing out significantly from other emotional states. Under these lighting conditions, participants were induced to experience the most positive and pleasant emotional states.

By contrast, under 6500 K lighting, more complex variations occurred, with multiple emotions fluctuating noticeably. The “happy” state reached a maximum of 0.65, while “sad” and “surprise” showed marked increases, around 0.3 ± 0.2. Under red, blue, and green lighting, “sad” and “neutral” dominated, fluctuating widely between 0.2 and 0.8, whereas other emotions remained very low (below 0.1). Consequently, these lighting colors were identified as those most likely to evoke “sad” and least likely to elicit happiness.

These findings confirm that participants’ preliminary emotional fluctuation plots effectively reflect the intensity or likelihood of each emotion at different time points [47].

### 3.2. Lighting Color and Emotional States

Lighting can regulate human emotions and enhance comfort within specific spaces [48]. By analyzing control groups of consumer emotions under different lighting conditions, participants’ willingness to eat and liking levels across experiments with varying color temperatures were obtained, and their subjective preferences were further compared with facial recognition analysis results.

Through examination of participants’ facial expressions under different lighting colors, During training, F was kept fixed to prevent the FER model from overfitting to the training set. The FER model (e.g., ResNet-18) was trained to learn a mask for the extracted facial features. Features f $\in R^{N\times C\times 1\times 1}$ were obtained from the Global Average Pooling (GAP) layer after x, and resized to generate the facial feature mask $Μ\in R^{N\times C}$. Their corresponding emotional states and emotional intensities toward the food were identified. Subsequently, participants’ subjective preference ratings were statistically tested to analyze differences in liking and willingness to eat across lighting conditions. Finally, the facial expression analysis results were compared with subjective preference scores to further verify whether the two sets of experimental outcomes demonstrated consistency.

#### 3.2.1. Group Analysis of Lighting Color Effects on Emotional States

The collected facial emotional responses of consumers toward food under different lighting colors were analyzed. The correlation of each participant’s emotion reflected the proportion of between-group variance relative to the total variance. The results indicated that only the “happy” emotion was significantly influenced by lighting color.

**Figure 5:**
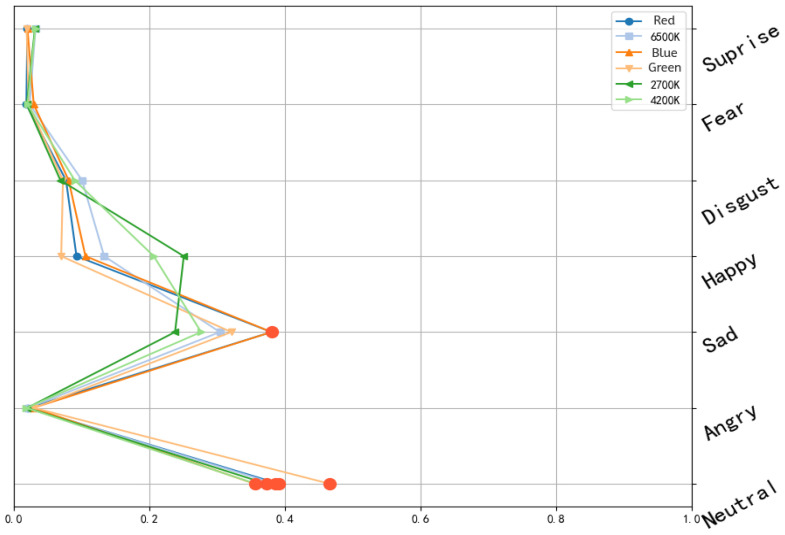
Average emotional intensity under different lighting conditions (The orange dots represent the maximum value of each broken line).

Under 2700 K warm white light, the mean intensity of “happy” was 0.25 (SD = 0.12), indicating a clear positive affect. The mean intensity of the “neutral” expression was 0.38, suggesting relatively stable emotions. Under 4200 K neutral white light, the mean intensity of “happy” was 0.21, representing a moderately positive affect, while the mean intensity of “neutral” was 0.36, lower than the average neutral intensity under other lighting conditions. This suggests that this lighting did not impose strong visual or emotional stimulation. Under 6500 K cool white light, the mean intensity of “sad” was 0.30, relatively higher than under warm tones, possibly due to the cooler lighting characteristics suppressing the generation of positive emotions (Figure 5).

The effects of red light on emotions were more pronounced. Under red illumination, participants’ mean intensity of “happy” dropped to 0.08, while “sad” increased to 0.38. Consumers tended to be more easily stimulated under red light, and thus this color should be used cautiously in external environments, as it may cause tension or stress [48].

Under green light, the mean intensity of “sad” was relatively high (0.31), ranking among the highest across lighting conditions. This may be because, in some consumers’ cognition, green can be associated with negative natural phenomena such as mold or spoilage, thereby triggering negative emotions. Under blue light, participants exhibited the highest mean intensity of “sad” across all lighting types (0.39). Meanwhile, the mean intensity of “happy” was slightly lower, and the mean intensity of “fear” was somewhat elevated.

In summary, the facial expressions elicited by food under specific lighting conditions exhibited distinct characteristics. Although subtle variations existed among these expressions, they could not account for the majority of variability in consumers’ facial responses. Nevertheless, the experiment demonstrated that “happy” was the primary emotion distinguishing food preferences. A higher level of pleasure was often accompanied by lower levels of negative emotions associated with the food. Moreover, the visual presentation of food under different lighting conditions exerted a significant impact on consumers’ taste perception and emotional experience [49]. The experimental findings further confirmed the following hypothesis: that controlling lighting color variations induced noticeable emotional fluctuations in participants across different lighting environments.

In addition, consumers exhibited generally higher acceptance of food under warm daylight illumination (2700 K) compared with cool blue light conditions. As reflected in participants’ observed emotions, warm daylight lighting on food enhanced its perceived sensory value. However, it is noteworthy that the uniqueness of the results was relatively low, and the variability of consumer emotions under identical lighting conditions should be further examined. A clear correlation exists between consumers’ psychological factors and food preferences [50]. Importantly, participants’ emotional states only influenced responses of the same valence: for instance, positive states were only correlated with positive emotions, while positive emotions could not reduce negative responses evoked by food [51].

Therefore, in the overall comparison of experimental results, the distribution of all participants’ emotional states was visualized, and the average intensity of positive and negative emotions under each lighting condition was represented in aggregated charts.

#### 3.2.2. Subjective Preferences: Eating Willingness and Liking

**Figure 6:**
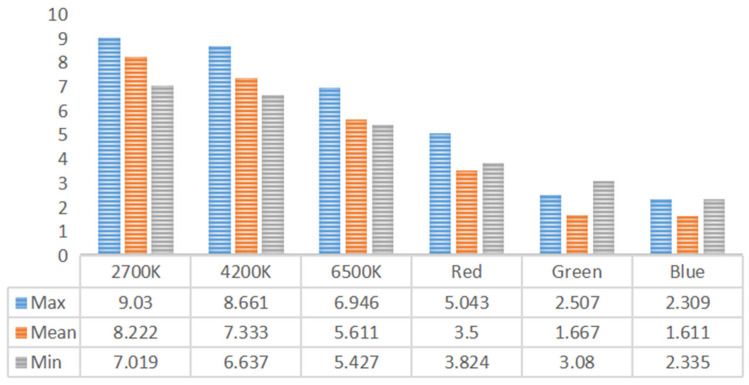
Average liking differences across six lighting conditions.

As shown in Figure 6, the interaction effect between fried chicken with French fries and lighting color on consumers’ appearance preference was significant [S-W test: F = 0.903, *p* < 0.001]. Consumers’ liking of food appearance under 2700 K and 4200 K lighting was significantly higher than under green and blue lighting (all comparisons, *p* < 0.001).

Specifically, food appearance received the highest liking scores under 2700 K and 4200 K warm light conditions (all comparisons, *p* < 0.001), whereas it received the lowest scores under green and blue lighting (all comparisons, *p* < 0.05). Overall, consumers’ appearance preference for food was significantly higher under 2700 K and 4200 K lighting than under blue and green lighting (all comparisons, *p* < 0.001).

**Figure 7:**
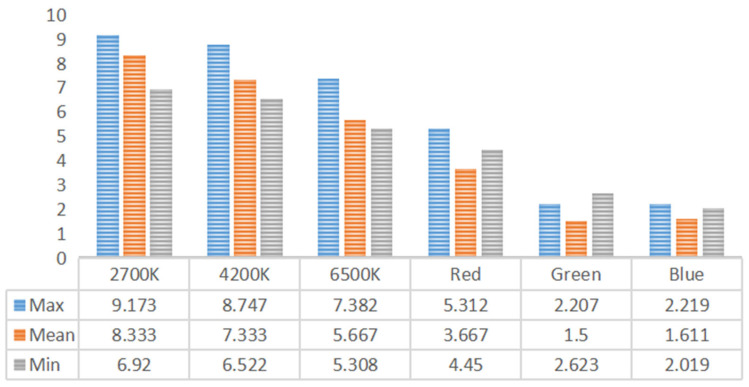
Average willingness differences across six lighting conditions.

As shown in Figure 7, the interaction effect between fried chicken with French fries and lighting color on consumers’ willingness to eat was significant [S-W test: F = 0.893, *p* < 0.001]. Participants under 2700 K and 4200 K lighting conditions were significantly more willing to choose fried chicken with French fries than under green and blue lighting (all comparisons, *p* < 0.05).

Willingness to eat was lowest under green lighting (for all comparisons, at least *p* < 0.001). For fried chicken with French fries, participants showed the highest willingness to eat under 2700 K warm light, whereas green lighting (all comparisons, at least *p* < 0.001) and blue lighting (all comparisons, *p* < 0.001) produced the lowest willingness levels. Participants’ willingness to eat under green and blue lighting was significantly lower than under 2700 K and 4200 K lighting (all comparisons, *p* < 0.001). Under 6500 K lighting, participants’ willingness to eat showed no significant differences (all comparisons, *p* < 0.05).

The interaction between consumers’ liking of food appearance and their willingness to eat was significant. Under 2700 K warm lighting, participants exhibited greater willingness to eat compared with green and blue lighting. Since the food samples were yellow in color, green lighting reduced participants’ willingness to eat to some extent, as illustrated in Figure 6 and Figure 7. Considering that yellow and blue are complementary colors, the complementary relationship between colored lighting and food color not only decreased consumers’ liking of food appearance but also reduced their willingness to eat. For example, light orange lighting makes cucumbers most attractive, whereas light blue lighting is most suitable for eggplants, highlighting that different foods are associated with different ambient lighting conditions [52].

Therefore, this experiment verified the following hypothesis: lighting color can modulate consumers’ preference choices and significantly influence both their willingness to eat and their liking of food appearance.

#### 3.2.3. Correlation Between Facial Expression Recognition and Subjective Ratings

The study analyzed participants’ subjective preference scores regarding liking and willingness to consume the food samples under different lighting conditions, along with the emotion recognition data obtained from facial expression analysis under the same conditions. A correlation analysis was further conducted to explore the relationship between participants’ subjective preference scores and objectively recognized emotions. If participants gave higher preference scores while simultaneously displaying more positive emotions such as “happy” or satisfaction on their faces, a strong positive correlation was likely to emerge. Emotional states play an important role in affective evaluation and are closely linked to consumers’ purchase intentions. Consumers are more willing to pay higher prices for foods that elicit positive emotions, whereas negative emotions tend to reduce willingness to pay [53].

A paired-sample test (S-W test) was conducted to determine whether significant differences existed between consumers’ subjective preference scores and the objective intensity values of their emotions. The results indicated that although an overall positive correlation was observed, significant discrepancies arose in certain cases. For example, under 6500 K lighting conditions, participants might subjectively report relatively high preference scores, while their facial expressions objectively indicated lower positive emotional intensity. This inconsistency may be attributed to social desirability bias in self-reports, where participants overestimated their preferences to align with perceived expectations.

Consumers’ liking of food appearance was highest under 2700 K warm white lighting (mean = 8.222), followed by 4200 K neutral white lighting (mean = 7.333). By contrast, the lowest scores were recorded under green (mean = 1.667) and blue (mean = 1.611) lighting. This suggests that participants liked the food’s appearance more under warm white light and less under green and blue light. A similar trend was observed for willingness to eat, with the highest average score under 2700 K warm white lighting (mean = 8.333) and the lowest under green (mean = 1.5) and blue (mean = 1.611) lighting, indicating that warm white light enhanced willingness to eat, whereas green and blue light were less effective.

Under 2700 K warm white lighting, the average intensity of “happy” identified by facial expression recognition ranged from 0.2 to 0.3. Subjective preference scores for food appearance averaged 8.222, and willingness to eat averaged 8.333. This demonstrates a strong positive correlation between objective positive emotional intensity and subjective liking/willingness under this lighting condition. Conversely, under green lighting, participants exhibited relatively higher average levels of “disgust” and “sad.” In the subjective preference ratings, the average liking for the appearance of the food was 1.667, and the average willingness to eat was 1.5. This indicates a clear correspondence between objectively evoked negative emotions and subjectively low food preference. The decline in subjective preference exceeded the increase in negative emotional intensity, suggesting that green light exerted a significant negative influence on consumer perception.

Under red lighting, participants showed the strongest intensity of “sad” emotion, yet their subjective preference scores were 3.5 for appearance and 3.8 for willingness to eat. Although red lighting objectively evoked higher levels of sadness, its negative effect was not as extreme in subjective ratings. This suggests that factors such as inherent liking for the food or hunger may have buffered the negative emotional impact of the lighting environment. Moreover, consumers may have judged the food samples based on preconceived notions or opinions even before testing, exaggerating positive attitudes while underestimating negative ones in self-reported surveys [54].

The comparative analysis supported the following hypothesis: in food consumption contexts, consumers’ facial expressions can reflect both the type and intensity of their emotional states, and these are positively correlated with the results of subjective preference surveys.

### 3.3. Variability of Emotional States and Eating Willingness the Experiment Identified Consumer

Emotions associated with food images by integrating computer vision techniques with psychological principles of emotion, perception, and affective interaction, thereby achieving an inclusive perspective on the “food–emotion relationship” [55]. By comparing participants’ emotional states under different conditions (e.g., various lighting colors), the variability of emotional states was analyzed. For instance, if the emotional centroid under red lighting exhibited significantly higher arousal than under blue lighting, it indicated that red light enhanced participants’ emotional arousal. Compared with subjective self-reports, physiological measures such as facial expression recognition can capture additional information to better understand the dynamics of consumer emotional responses [56].

Regarding the fluctuation ranges of participants’ emotional intensity, under 2700 K warm white lighting, the intensity of the “happy” emotion exhibited minimal variation, suggesting that participants’ overall positive emotional intensity was consistently high. In sharp contrast, under red lighting, the mean intensity of “sad” reached as high as 0.39, but the fluctuation range was large, indicating pronounced individual differences in sadness. This was likely due to varying degrees of sensitivity to red light among consumers, resulting in divergent emotional responses.

According to the trend of participants’ emotional intensity, as shown in Figure A1. As the color temperature increased from 2700 K to 6500 K, the intensity of “happy” first rose slightly (peaking at 0.79 under 4200 K) before dropping sharply (down to 0.48 under 6500 K). Meanwhile, the intensity of the “neutral” expression steadily increased, from 0.69 at 2700 K to 0.71 at 6500 K. This indicates that low color temperature warm white light more effectively elicited positive emotions, whereas higher color temperatures, with cooler lighting, gradually shifted participants’ emotions toward neutrality.

Under blue lighting, participants’ intensities of “disgust” and “sad” were significantly higher than under other lighting conditions. This strongly confirmed the intrinsic association between blue light and negative consumer emotions. These findings further supported hypothesis, demonstrating that different lighting colors exert significant effects on consumers’ emotional perception.

### 3.4. Influence of Lighting on Eating Willingness

Combining facial expression recognition with subjective evaluation methods provides a more effective assessment of consumers’ acceptance of products [21]. The experimental results showed that under red lighting, participants exhibited higher levels of pleasure and willingness to eat, whereas blue lighting reduced these indicators. Green lighting also had predominantly negative effects on consumers’ emotional perception, while cool white lighting resembled the neutral condition.

Facial expression analysis revealed specific facial action units associated with changes in consumers’ emotional states, further validating the subjective Likert-scale ratings. Under 2700 K and 4200 K lighting, participants’ “happy” emotions increased significantly, and subjective preference surveys likewise indicated positive tendencies, demonstrating a high degree of consistency between objective and subjective findings. By contrast, under blue and green lighting, facial expression analysis revealed strong “sad” emotions, which corresponded to the lowest levels of pleasure and willingness in subjective ratings. The data thus confirmed the consistency between facial recognition and subjective preferences.

It is noteworthy that under red lighting, consumers’ pleasure and willingness to eat appeared in a “neutral” state, while facial recognition analysis indicated high intensities of both “sad” and “neutral”. This result showed relatively low consistency with subjective ratings. Some participants, possibly due to personal experiences or heightened visual sensitivity to red light, expressed extremely negative evaluations of fried chicken with French fries. Others, however, were buffered by additional factors such as strong liking for the food, which diminished the negative effect of red lighting, leading to more positive evaluations. This further suggests that results obtained through facial expression recognition exhibit higher sensitivity compared with Likert-scale evaluations [32].

The experiment demonstrated that food itself can stimulate consumer emotions, partially inducing emotional responses that may affect accurate estimation of consumers’ subjective–physiological emotional coherence [57]. Food preferences are subject to change as consumers’ perceptions, expectations, and prior appetite states evolve [58]. In the experiment, consumer emotional responses induced by food were averaged across repeated exposures to the samples and color lighting conditions. No systematic changes in participants’ facial expressions were observed after repeated food exposure (no significant main or interaction effects). Consequently, during repeated exposure, the “neutral” emotional state appeared frequently, thereby reducing the interaction between consumers, colored lighting, and food.

## 4. Discussion

### 4.1. Correlation Between Facial Emotional Responses and Subjective Preferences

Based on the experimental data from ‘18 participants + 6 lighting conditions’, the study can preliminarily determine whether a particular lighting condition generally triggers positive emotions in participants or whether there are significant individual differences (e.g., some individuals are sensitive to blue light, while others show no significant response). This ‘qualitative + quantitative’ preliminary observation can provide a basis for subsequent research to identify ‘individual variables that need to be controlled’ (e.g., whether to group by visual sensitivity, age differences, dietary preference differences, etc. The experiment demonstrated that different lighting colors can alter consumers’ liking and acceptance of food. A significant interaction was found between food samples and lighting colors. Warm white lighting increased consumers’ liking of the appearance of fried chicken with French fries and their willingness to eat, whereas green and red lighting had a substantially negative impact on both indicators. Interestingly, although red light is a warm color, it elicited relatively negative emotions, which may be attributed to associations triggered by the color red. Similarly, specific colors appear to guide implicit evaluations of food, with red functioning as a subtle avoidance cue in food contexts [59]. Compared with fluorescent and metal halide lighting, incandescent lighting rendered the color of fried chicken with French fries more natural, leading more consumers to prefer the food under this illumination [16]. The experiment also showed that there was no significant difference between 2700 K and 4200 K warm lighting sources, both producing a positive influence. This result may be explained by the intrinsic relationship between the food’s color and the lighting color: fried chicken with French fries has a warm yellow hue as its basic chromatic attribute, and thus warm white light at 2700 K and 4200 K does not produce abrupt emotional preference responses.

In terms of relative brightness differences across light sources, because fluorescent lamps and metal halide lamps emit little red light, they do not enhance color preference for red foods (e.g., red bell peppers). However, for yellow foods (e.g., yellow bell peppers), no significant differences in color preference were observed across fluorescent, metal halide, and incandescent lighting, since all three emit strong yellow light.

Moreover, lighting color and brightness can regulate consumers’ emotional states [16]. Given that consumers’ emotions influence food choice and intake, the emotional states induced by different lighting colors can alter their liking of food appearance and willingness to eat fried chicken with French fries [60]. However, previous studies have not consistently observed such relationships between food choice and emotional changes. Many factors may shape consumers’ willingness to eat and their choices, including the intensity of emotional influence, the relevance of emotions to food, dietary control, and emotional eating behaviors, all of which may drive food selection and intake [60].

From the experimental results, higher emotional arousal among consumers appeared to be associated with stronger negative emotions. Specifically, as the arousal (i.e., intensity) of emotions increased, willingness to eat decreased, suppressing food desire. Furthermore, since participants evaluated fried chicken with French fries under different lighting conditions while being directly exposed to the illumination, this maximized the impact of lighting on emotional changes in the study.

The sensory attributes of food—such as its visual, olfactory, and tactile qualities—can all elicit direct emotional responses from consumers. For instance, consumers may feel bored by bland snacks, surprised by exotic fruit flavor–color combinations, or amused by the texture of cotton candy [61]. When negative emotions are triggered, restrictive eaters and non-restrictive eaters differ significantly in their evaluations of high-calorie foods. This suggests that emotional states may modulate individuals’ preferences and evaluations of food. This finding aligns with the observed positive correlation between facial emotional responses and subjective food preference ratings in the present study. Moreover, it is consistent with prior literature emphasizing the positive correlation between high-calorie food preferences and consumers’ positive emotional responses.

### 4.2. Cross-Validation of Facial Recognition and Subjective Ratings

The study assessed participants’ explicit subjective preferences for high-calorie foods from 18 individuals and combined them with implicit data from facial expression recognition to examine whether a positive correlation existed between the two. During consumer evaluations, rapidly changing emotional information was captured. By combining subjective preference measurements with response detection through facial analysis, mutual verification of subjective and objective results was achieved [39]. While many consumers restrained themselves in emotional expression, spontaneous responses also occurred. Differences between reported emotions and facial expressions may be attributable to individual differences among participants. The results indicated that self-reported food-evoked emotions and facial expression recognition reflected different aspects of food influence. For example, self-reported emotions may be more closely related to the hedonic aspects of food, whereas facial expression recognition (and implicit measures in general) may capture other aspects of food evaluation. Consumer emotional expressions show significant individual differences. Some participants exhibit restraint in emotional expression, while others show spontaneous reactions. This difference may be related to baseline preferences and potential cultural backgrounds; for example, participants with a high initial preference for fried chicken and fries show stronger consistency between their self-reported enjoyment ratings and facial expressions of positive emotions, while those with a low baseline preference show more noticeable differences between these two measurements. Further analysis reveals that self-reported emotions may be more closely related to the hedonic experience of food (e.g., ‘Is it tasty?’), while implicit measurements such as facial expression recognition may be more sensitive to other potential influences on food perception (e.g., subconscious food safety perceptions triggered by lighting color).

Emotions are essentially non-cognitive, making them difficult to capture, measure, and quantify. Current tools for measuring emotions include physiological methods, facial recognition, verbal self-report, and visual self-report [62]. For instance, studies using ERP (event-related potentials) have revealed how food liking influences brain responses and functional connectivity, particularly under visual food stimulation [7]. These findings highlight the strong connection between subjective food preference ratings and physiological emotional responses. A prior attentional bias study also showed that negative emotions increase consumers’ attentional bias toward food cues and subjective appetite, with a positive correlation between the two, suggesting a shared mechanism of food reward system activation. Moreover, attentional bias has been linked to dietary traits such as external and restrained eating, indicating that current emotions and individual eating styles jointly affect food motivation [63]. Similarly, research capturing subjective ratings and physiological signals during food consumption showed that subjective ratings of flavored gel foods were negatively correlated with facial EMG activity of the corrugator supercilii, and positively correlated with EMG activity of the masseter and suprahyoid muscles [64].

The study preliminarily concluded that lighting color can modulate food perception and consumption motivation. It also found a positive correlation between consumers’ subjective preference surveys and facial expression recognition results. The study supports and extends the concept that lighting color can regulate food perception and consumption motivation. It confirmed the positive correlation between subjective preference surveys and facial expression recognition. Under controlled conditions using LED lighting, lighting color influenced not only liking of food appearance but also appetite. Furthermore, participants’ subjective preferences were positively correlated with emotional perceptions induced by exposure to experimental lighting. Based on these results, consumer purchase intentions may increase when certain types of foods are displayed under specific lighting colors in retail environments. For example, one of the simplest and most effective ways to attract customer attention during food choice and purchase is through visually striking product lighting [65]. Different fluorescent lamp wavelengths under varying lighting conditions significantly affect food color fading. Since fluorescent lighting is commonly used in vegetable retail environments, it may influence the perceived color quality of processed foods and thereby affect consumer purchasing choices [66]. The development of LED lighting has made illumination for meat displays increasingly important [67]. If a certain lighting color is associated with higher preference scores and positive emotions, restaurants may consider using such lighting in dining areas to enhance the customer dining experience. Correlations between emotions and sensory attributes can also aid food innovators in introducing promising products in competitive markets [68].

The experiment provided a preliminary understanding of how different lighting conditions can trigger different emotional responses in individuals by analyzing emotional data under various lighting conditions. It also offered a solid foundation for comparing facial expression data with subjective preference scores. By comparing objective emotional data from Generalizable Facial Expression Recognition (GFER) with subjective ratings, the study further revealed the complex relationship between lighting-induced consumer emotions and food-related preferences [69]. Consumers’ facial expressions can distinguish between stimuli of different intensities and hedonic levels [70]. The experiment combined research on lighting and emotional perception with existing psychological theories such as cognitive appraisal theory and emotional contagion theory. From the perspective of cognitive appraisal theory, it analyzes how different lighting environments may influence consumers’ cognitive evaluation processes of sample foods, thereby affecting their emotional perceptions.

### 4.3. Limitations of Facial Expression Recognition Technology and Subjective Preference Rating Validation

This study also has certain limitations. First, the sample size of the experiment is relatively small. As a preliminary exploration, the 108 sets of data (18 participants × 6 lighting conditions) only reflect trend associations and do not allow for complex interaction effect analysis. Future research, after expanding the sample size (suggested ≥100 participants), can further verify the moderating effects of variables such as age and gender. Due to the limited sample size and the social and cultural backgrounds of the participants, the interpretation of our findings should be cautious. It would be insightful to see if the same results can be found in individuals with different backgrounds. Future research could validate the generalizability of the results by increasing the sample size.

Second, the study only used brown food. Future research could introduce foods of different colors to compare the effects of different colors on emotions and appetite. Foods of different colors (such as red strawberries, green vegetables, white dairy products, black chocolate, etc.) may form “synergistic” or “conflict” effects with lighting colors. The color of the food itself carries appetite signals, and lighting color may amplify or weaken this signal. Introducing multi-colored foods could explore the contributions of both “natural food color” and “lighting intervention” to appetite, revealing more detailed mechanisms of influence.

Third, there are limitations in the facial expression classification method. In addition to the existing facial expression recognition (GFER) data and subjective preference ratings, it may be useful to incorporate more physiological indicators, such as heart rate, skin conductance, etc. For example, by measuring consumers’ heart rate changes when viewing fried chicken and fries under different lighting, if the heart rate significantly increases under red light, it could indicate that the lighting triggered a stronger emotional arousal, further confirming the impact of light on emotions. At the same time, combining EEG (electroencephalography) data to analyze the activity in different brain regions could help explore the neural mechanisms of how light affects emotional perception at the brain level, such as whether certain lighting activates brain areas related to emotional processing. Conducting dynamic emotional analysis would not only focus on the emotional intensity at a particular moment but also analyze the dynamic changes in emotions over time.

Fourth, cultural and individual difference factors still need to be further measured. Consumers’ perceptions of colors can vary significantly across different cultural backgrounds. For example, green is often seen as a symbol of decay and staleness in some cultures, while in others, it represents freshness and growth. These cultural differences may have a significant impact on consumers’ emotions and food choices, especially under varying lighting conditions. This suggests that color perception is not merely a visual response but is also deeply influenced by personal experiences and cultural background.

Such cultural and individual differences may be specific to the type of food being tested. For example, the brownish-yellow, high-calorie foods used in this study (such as chicken and fries) may evoke different emotional responses across cultures. In some cultures, these foods may be associated with high calories, comfort, and festive atmospheres, while in others, they might be viewed as unhealthy options, thereby affecting consumers’ appetite and willingness to eat. Therefore, future research could further validate this phenomenon by selecting different types of food, exploring the profound influence of different cultural and individual preferences on food perception.

## 5. Conclusions

This study systematically explored consumers’ food preferences and emotional perceptions under different lighting colors. By employing the Likert scale, chi-square tests, ANOVA, and facial expression recognition, data were collected and analyzed from 18 participants. The results demonstrated that lighting color influences consumers’ food preference choices and induces different emotional states. Under 2700 K warm white lighting, participants exhibited stronger appetite, whereas green and blue lighting elicited lower willingness to eat. Subjective preference scores provided direct results of consumers’ conscious evaluations, while facial expression recognition data captured subconscious emotional responses. Furthermore, the analysis of consumers’ facial expressions was positively correlated with self-reported results. The study supports and extends the notion that lighting color can modulate food perception and eating intention, confirming that different lighting colors are key factors affecting consumers’ food choice behavior and emotional feedback.

Nevertheless, the study has certain limitations. Due to the relatively small sample size and the social and cultural backgrounds of participants, the experimental population requires further expansion. The current sample size of 18 participants can only provide preliminary insights, and the findings need to be validated in larger, more diverse samples. Future research should also include a broader range of food types to explore whether different foods, with varying colors and characteristics, produce similar or different emotional and preference responses under various lighting conditions. This would help refine the conclusions and ensure their broader applicability.

## Figures and Tables

**Figure A2:**
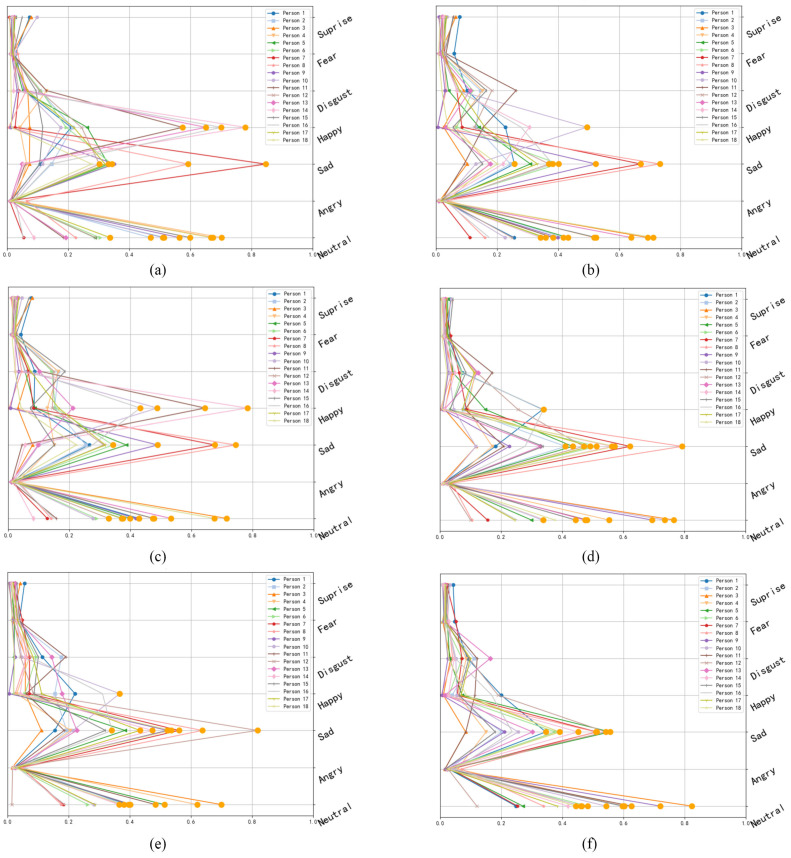
Individual emotional intensity under different lighting conditions ((**a**): 2700 K; (**b**): 6500 K; (**c**): 4200 K; (**d**): red; (**e**): blue; (**f**): green; The yellow dots represent the maximum value of each broken line).

## Data Availability

The original contributions presented in this study are included in the article. Further inquiries can be directed to the corresponding author.

## Appendix A

### Appendix A.1

The figure presents the fluctuation curves of participants’ preliminary emotional changes under the six lighting conditions.

### Appendix A.2

The figure is the trend of participants’ emotional intensity. By comparing participants’ emotional states under different conditions (e.g., various lighting colors), the variability of emotional states was analyzed.
